# Cardiac MRI and Myocardial Injury in COVID-19: Diagnosis, Risk Stratification and Prognosis

**DOI:** 10.3390/diagnostics11010130

**Published:** 2021-01-15

**Authors:** Saagar K. Sanghvi, Logan S. Schwarzman, Noreen T. Nazir

**Affiliations:** 1 Division of Cardiology, University of Illinois College of Medicine at Chicago, Chicago, IL 60612, USA; ssangh8@uic.edu; 2 Department of Medicine, University of Illinois College of Medicine at Chicago, Chicago, IL 60612, USA; schwar37@uic.edu

**Keywords:** COVID-19, cardiac magnetic resonance imaging (CMR), myocardial inflammation, cytokine storm

## Abstract

Myocardial injury is a common complication of the COVID-19 illness and is associated with a worsened prognosis. Systemic hyperinflammation seen in the advanced stage of COVID-19 likely contributes to myocardial injury. Cardiac magnetic resonance imaging (CMR) is the preferred imaging modality for non-invasive evaluation in acute myocarditis, enabling risk stratification and prognostication. Modified scanning protocols in the pandemic setting reduce risk of exposure while providing critical data regarding cardiac tissue inflammation and fibrosis, chamber remodeling, and contractile function. The growing use of CMR in clinical practice to assess myocardial injury will improve understanding of the acute and chronic sequelae of myocardial inflammation from various pathological etiologies.

## 1. Introduction

The worldwide coronavirus disease2019 (COVID-19) pandemic has overwhelmed healthcare systems across the world since the outbreak began in Wuhan, China, in December 2019. The number of individuals infected along with mortality from the disease is growing exponentially in several regions of the world [1]. Beyond respiratory decompensation, the SARS-CoV-2 virus has been shown to cause a prolonged disease course frequently complicated by severe systemic hyperinflammatory disease. Myocardial injury as evidenced by clinical, laboratory, and diagnostic evaluation has also been observed in the advanced stages of the COVID-19 infection.

Cardiac MRI (CMR) has evolved to become the preferred imaging modality for non-invasive evaluation in acute myocarditis [2,3], as pathognomonic features of myocardial inflammation can be identified with improved sensitivity and specificity [4]. T2-weighted imaging, early gadolinium enhancement (EGE), and late gadolinium enhancement (LGE) signal intensities used to see pathophysiologic changes from edemas and hyperemia to fibrosis are coupled with quantitative parametric mapping of T1 and T2 relaxation times to improve diagnostic accuracy and identify both acute and chronic changes of myocardial inflammation. In a COVID-19-induced myocardial injury, CMR can assess the extent, chronicity, and severity thereof. Tissue characterization with CMR is critical to evaluating prognosis in the acute and chronic phases of the myocardial and systemic disease course [4,5].

## 2. Myocardial Injury in COVID-19

### 2.1. Incidence and Significance

Myocardial injury is not uncommon in the COVID-19 patient population. Retrospective study data from various sources internationally suggest 20–30% of COVID-19 cases will have signs of acute myocardial damage [6,7,8]. A Lancet study from February 2020 reviewing 52 critically ill patients from Wuhan, China, with COVID-19 showed myocardial injury, as indicated by high-sensitivity troponin greater than 28 ng/L in 29% of thde patients [6]. A JAMA study of 416 COVID-19 patients showed 20% with myocardial injury, as indicated by troponin I greater than 0.04 ng/mL [7]. A retrospective study analyzing 2253 patients (1002 “severe” cases) showed a higher prevalence of myocardial injury among the patients who died compared to the survivors (75.8% vs 9.7%, *p* < 0.001) [8]. Patients with myocardial injury were also shown to have a more severe acute illness, as indicated by higher inflammatory markers such as C-reactive protein, higher serum creatinine, more pulmonary infiltrates and opacities on chest imaging, and greater rates of non-invasive and invasive ventilation. Furthermore, elderly patients with comorbidities such as coronary artery disease, hypertension, and diabetes and chronic kidney disease were more likely to have a greater severity of illness [7]. Elevated troponin and absolute magnitude of elevation have also been associated with illness severity, increased rates of intensive care unit admission, and overall mortality [8,9,10,11,12,13,14].

Clinical indicators of myocardial damage include elevated cardiac biomarkers such as troponin I, ST segment and T wave changes on electrocardiogram (ECG), and newly reduced left ventricular ejection fraction (LVEF) on cardiac imaging. COVID-19 patients have demonstrated a propensity for marked troponin elevations commonly seen in patients with acute coronary syndrome; however, COVID-19 patients are frequently found without obstructive epicardial coronary artery disease on angiography [14]. This suggests either direct myocardial injury damage caused by an infiltrating SARS-CoV-2 virus or indirect mechanisms of inflammatory myocardial injury secondary to the viral infection. Myocarditis has been well-documented in non-SARS coronavirus infections and previous epidemics of the coronavirus, including the Middle East respiratory syndrome and SARS-CoV-1 [14]. Notably, clinical signs of new-onset heart failure and cases of new-onset arrhythmia have been commonly observed with COVID-19. Data are relatively limited on SARS-CoV-2 myocarditis and typically confined to case reports of reduced LVEF and elevated troponin [7,9,10,11,12,13,14].

### 2.2. Mechanisms of Myocardial Injury

Various mechanisms of myocardial injury in COVID-19 have been proposed: direct viral injury causing myocarditis, systemic inflammation secondary to cytokine storm, stress-induced cardiomyopathy, microvascular thrombosis, among others [14,15,16,17,18,19]. In the typical viral myocarditis pathophysiology, entry of viral particles in cardiac myocytes leads to an innate and humeral immune response and results in focal or diffuse myocardial necrosis. Within a few days of a direct cellular injury, edemas and necrosis can lead to clinical symptoms associated with adverse changes in myocardial contractile function, including heart failure and arrhythmia.

In the COVID-19 pathophysiology, the SARS-CoV-2 viral S1 protein binds to the ACE-2 receptors on target host cells and, in conjunction with TMPRSS2 protease activity, gains host cell entry to initiate infection [20]; ACE-2 receptors are found on the respiratory and gastrointestinal tract epithelium and on cardiomyocytes. Interestingly, these receptors have been shown to be upregulated in the myocardium with heart failure or the use of ACE inhibitors, though an increased number of receptors has not correlated with greater viral cardiotropism [21]. Furthermore, the lifespan of this virus within the host is unclear; the SARS-CoV-1 virus has shown the ability to persist in the myocardium for several weeks to months, but has also been shown to clear the myocardium in less than a week. It is also unclear if SARS-CoV-1 myocarditis would lead to chronically dilated cardiomyopathy as with other more viral etiologies of myocarditis such as adenovirus or enterovirus [22]. 

### 2.3. Inflammation-Mediated Myocardial Injury

The pathophysiological mechanism of direct viral injury has yet to be proven in SARS-CoV-2. Autopsy studies of patients who died from the COVID-19 illness described SARS-CoV-2 within the alveolar tissue but not the cardiac tissue; however, evidence of myocardial cell degeneration and necrosis were seen [15]. Tavazzi et al. described a COVID-19 patient who developed primary cardiac decompensation and shock with markedly elevated troponin requiring venoarterial extracorporeal membrane oxygenation. Cardiac biopsy revealed viral particles within interstitial cell macrophages, but not within cardiac myocytes, suggesting an indirect inflammatory mechanism of myocardial injury [16].

Profound inflammatory responses with marked cytokine production in hospitalized patients with COVID-19 have been well-documented in the advanced stages of the disease [14,15,16,17,18,19]. This hyperinflammation stage, as described Siddiqi et al., is associated with myocardial injury and systemic decompensation and can occur sometimes more than 10 days after the onset of mild symptoms. This may contribute to the relative clinical stability seen in COVID patients prior to abrupt decompensation. The constellation of inflammatory mediators and cytokines seen in COVID-19 has similarities to chimeric antigen receptor T-induced cytokine release syndrome, which results in a marked inflammatory response in oncology patients treated for various types of leukemia and lymphoma [23]. This inflammatory cascade activates T cells and immune effector cells, including macrophages and endothelial cells, to produce inflammatory mediators, such as tumor necrosis factor and interleukin-6 (IL-6), associated with disseminated intravascular coagulation (DIC) and cardiomyopathy. In a retrospective study of 183 Chinese COVID-19 patients, coagulopathy was associated with high mortality and over 70% of the patients who died met criteria for DIC, suggesting microvascular thrombosis as a potential mechanism [17,18]. The importance of IL-6 to the cytokine storm cascade is shown by the often rapid abrogation of the syndrome by interruption of IL-6 signaling by receptor antagonist tocilizumab [22].

## 3. Cardiac MRI for Myocardial Injury in COVID-19

### 3.1. Acute Myocarditis Workup and CMR

Viral myocarditis can present with various symptoms: from mild atypical chest pain to symptoms mimicking acute coronary syndrome (ACS), such as crushing chest pain with dyspnea or palpitations. In COVID-19, myocardial injury is most commonly seen in the later hyperinflammatory stage, sometimes occurring several days after onset of initial infectious symptoms. Therefore, the symptoms and objective findings associated with myocardial inflammation can be missed if the medical team is not aware or the patient is not evaluated appropriately for evidence of myocardial injury and dysfunction.

Workup can reveal dynamic ST segment or T wave changes or arrhythmia on ECG, elevated cardiac biomarkers, and sometimes focal wall motion changes on transthoracic echocardiogram (TTE) [4]. The conduction abnormalities seen on ECG are non-specific and unable to adequately assess for myocarditis. TTE may be useful to evaluate new-onset or acutely worsened heart failure, primarily to rule out new valvular disease or change in ejection fraction; however, a significant wall motion abnormality associated with ACS can also be seen in myocarditis. Endomyocardial biopsy (EMB) has traditionally served to confirm diagnostic histopathological and molecular analysis with the ability to identify infectious etiology. EMB also maintains an American College of Cardiology (ACC) 1B recommendation for severe acute-onset heart failure with hemodynamic compromise, new ventricular arrhythmia with severe structural remodeling, new high-grade AV block, or failure to respond to medical therapy [24]. EMB still features an inherent risk of complications as an invasive procedure and requires expertise that may not be found at smaller medical centers. Furthermore, there is a significant risk of sampling error; however, this has been improved with preprocedural localization of inflammatory changes using CMR [25,26,27,28,29,30,31].

Cardiac MRI is unique in its ability to non-invasively identify myocardial edemas and myocardial necrosis, localize sites of inflammation, and assess severity of tissue damage [4]. CMR is currently the gold standard for non-invasive detection and exclusion of myocardial inflammation. This imaging modality is particularly beneficial for allowing evaluation of epicardial or pericardial regions outside of biopsy range and for enabling non-invasive tissue characterization and identification of pathognomonic features of myocardial inflammation. For these advantages in comparison to other diagnostic modalities, CMR has a class 1 indication in the current European Society of Cardiology (ESC) guidelines for assessment of myocarditis and myocardial storage diseases [32,33,34,35,36].

Cardiac MRI is able to visualize and characterize inflammatory tissue changes secondary to viral myocarditis. Diagnostic targets in this inflammatory cascade are used to assess tissue changes consistent with acute or chronic inflammation. The typical acute phase of viral myocarditis involves a combination of direct viral injury and subsequent host immunologic reaction—in some cases, the inflammatory process may persist, resulting in a chronic post-infectious myocarditis that can progress to dilated cardiomyopathy [28,37].

### 3.2. Pathophysiology of Myocarditis as Seen on CMR

Pathophysiological changes in myocarditis include intracellular and interstitial edemas, dilatation of the myocardial vascular bed, and resulting hyperemia and capillary leaks, myocyte cell membrane injury, and necrosis, inflammatory cell infiltration, and ultimately collagen deposition with formation of interstitial fibrosis and scars. Using the original 2009 LLC, myocarditis is typically visualized with T2-weighted imaging to target edemas, with early gadolinium enhancement (EGE) to target hyperemia and capillary leaks, and with late gadolinium enhancement (LGE) to detect scars and fibrosis, but also acute changes in the extracellular volume [38]. A meta-analysis of the 2009 LLC reported a pooled diagnostic accuracy of 83%, sensitivity of 80%, specificity of 87%, consistent with several other large meta-analyses evaluating diagnostic criteria [39,40].

The revised 2018 LLC incorporated more recently developed quantitative parametric mapping to quantify T1 and T2 relaxation times—complex measurements of magnetic tissue properties as influenced by tissue characteristics, environmental conditions, and analysis methods. This provides a quantitative method of assessing tissue composition and pathological changes [4]. T2 mapping is useful to rule out active inflammation with a sensitivity of 89% [41]. T1 relaxation time is sensitive to acute and chronic inflammation, resulting in T1 mapping’s high negative predictive value of 92% for detecting inflammation [39].

#### 3.2.1. Myocardial Edema

**Figure 1:**
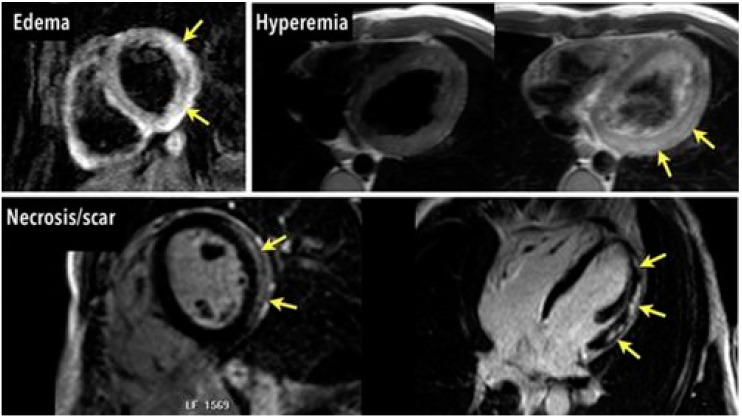
Myocarditis. Myocardial “Edema” identified using T2-weighted imaging. “Hyperemia” identified with early gadolinium enhancement. “Necrosis/scar” identified with late gadolinium enhancement. Yellow arrows are shown to indicate the respective re-gions of abnormal myocardial enhancement in myocarditis [45]

**Figure 2:**
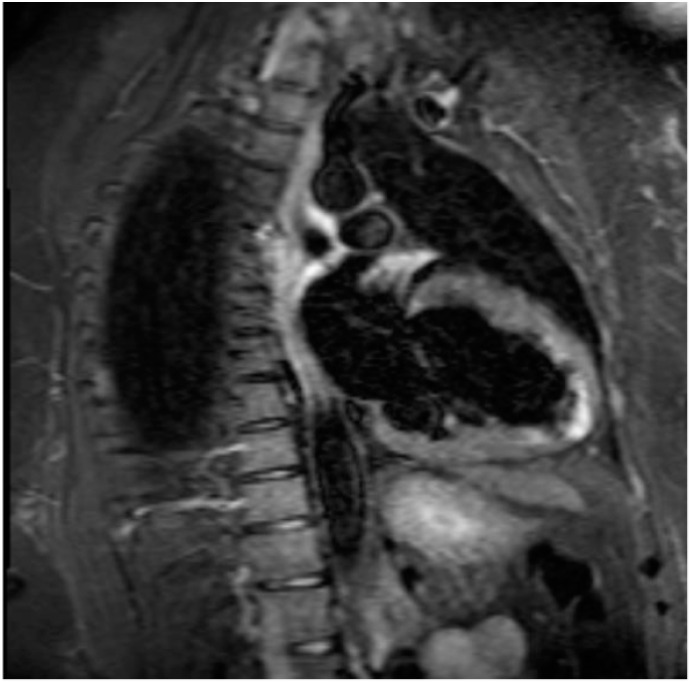
Regional myocardial edema in COVID-19—T2-weighted imaging.

Increased tissue water content caused by inflammation leads to the characteristic findings seen on CMR. Myocardial edema is seen as a regional or global hyperintensity on T2-weighted imaging (see Figure 1 and Figure 2). Water content causes a prolonged T2 relaxation time as measured with T2 mapping, which enables direct quantitative analysis of edemas. T1 relaxation time is also increased with an edema; however, more chronic tissue changes like fibrosis can also increase T1, making it less specific for myocardial edemas [42,43,44]. When a T2 signal intensity (SI) is unable to be localized, increased global SI can still be identified by taking a ratio of the myocardial SI referenced to skeletal muscle SI within the same image called the edema ratio. Previously established criteria across studies determined values above a threshold of 1.8 to 2.2 to be pathological [39]. Notably, the negative predictive value of T2-weighted imaging for acute myocarditis is shown to be 80% [39]. 

#### 3.2.2. Hyperemia and Extracellular Expansion

The inflammatory cascade can progress to tissue changes consistent with hyperemia, increased vascular permeability, and expansion of the extracellular space. A gadolinium-based contrast agent is visualized using T1-weighted spin echo imaging shortly after administration to acquire early gadolinium enhancement (EGE) images (see Figure 1). Theoretically, the increased extracellular space secondary to inflammation leads to hyperemia and a larger volume of distribution for gadolinium contrast and increased enhancement from contrast retention in the early washout period. Myocardial signal enhancement, defined as the percentage increase of SI with values above approximately 50% considered abnormal. Global relative enhancement is a ratio of myocardial SI referenced to skeletal muscle SI, with a value greater than 4.0 considered abnormal [46,47,48,49].

#### 3.2.3. Fibrosis and Scar Formation

**Figure 3:**
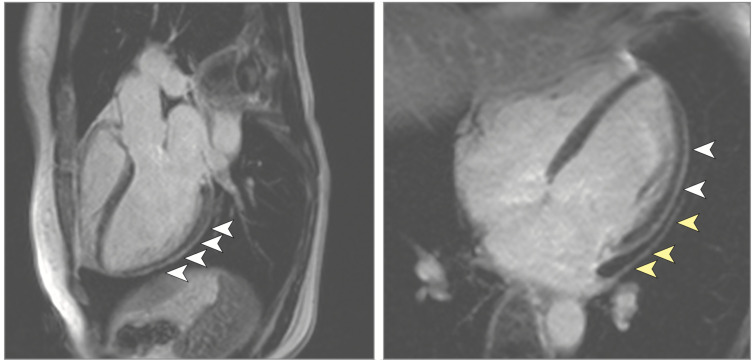
Pericardial effusion and enhancement (yellow arrowheads on the right image) and epicardial and intramyocardial enhancement (white arrowheads) in COVID-19, using late gadolinium enhancement [55].

Quantification of late gadolinium enhancement (LGE) has emerged as standard protocol in CMR to measure areas of infarction in ischemic heart disease and for risk stratification in ischemic (ICM) and nonischemic cardiomyopathy (NICM) [4,50] (see Figure 1 and Figure 3). Even though necrosis and fibrosis are commonly associated with LGE, it is important to note that LGE can represent one of four possible etiologies, all of which cause increases in ECV: myocardial inflammation, myocardial extracellular infiltration, acute myocardial necrosis, myocardial fibrosis. In cell necrosis and death, gadolinium is able to access the intracellular space of damaged myocytes; LGE is seen after the contrast agent has been allowed to wash out for at least 10–15 min. The LGE uptake lends to characteristic scar patterns seen in various NICM, as myocarditis typically leads to a diffuse, patchy, subepicardial pattern of LGE, typically with primary involvement of the lateral and inferolateral walls of the left ventricle. The infecting pathogen may also alter the distribution of LGE, as parvovirus B19 may be associated with subepicardial lateral wall LGE, while human herpesvirus 6 may be associated with mid-wall septal LGE [51]. Over time, with contraction of the scar tissue, the overall area of LGE will decrease, but the signal intensity will likely increase. In acute myocarditis, the presence of LGE is associated with worsened outcomes, and findings of edemas without LGE have been associated with improved recovery and prognosis [52,53,54]. Notably, acute versus chronic myocarditis cannot be differentiated with LGE alone. Furthermore, mild cases can be missed if concurrent T2 signal evaluation or T1 and T2 parametric mapping looking for edemas and hyperemia is not used as per the revised 2018 LLC. NICM can be differentiated from an ICM pattern which has predominant involvement of the subendocardium.

### 3.3. Chronic Myocarditis and CMR

**Figure 4:**
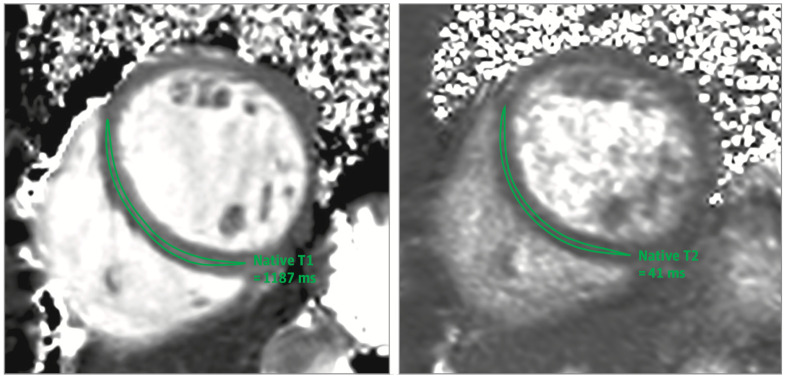
Quantitative parametric myocardial T1 and T2 mapping in COVID-19 showing significantly raised native T1 and native T2 in an adult woman with acute COVID-19 perimyocarditis. In this study, abnormal native T1 and T2 values were defined as greater than 1105 ms and greater than 37.4 ms, respectively, based on previously derived sequence-specific cutoffs of 2 SDs above the respective means in a healthy population [55].

CMR imaging can provide not only diagnosis of acute myocarditis, but also critical prognostic information, as the development of fibrotic tissue or chronic dilated cardiomyopathy can lead to worsened outcomes [4,50]. While there is decreased sensitivity for the diagnosis of chronic myocarditis after eight weeks from symptom onset, parametric mapping may be advantageous as it is not reliant on reference tissue imaging (see Figure 4). Previous studies have shown that the magnitude of cardiac biomarker elevation alone is independent of long-term outcomes in myocarditis, such as normalization of left ventricular function, progression to heart failure, or death [56,57,58]. 

Though the tracking and normalization of inflammatory markers have been associated with the resolution of myocardial inflammation, most recent CMR data have shown that ongoing inflammation may still be present. In a retrospective study published by the AHA, 71% of the patients had persistent LGE at the 3-month follow-up visit despite 88% of the patients showing normalization of cardiac and inflammatory biomarkers. Interestingly, the absolute level of biomarker elevation did not predict the presence or change in LGE on follow-up [59]. Barone–Rochette et al. found a position correlation between LGE extent at 3 months and adverse cardiovascular outcomes, including recurrent myocarditis, heart transplant, and all-cause mortality [54]. LGE is unable to differentiate persistent inflammation from fibrotic scars [38]. FDG PET/MR may have incremental value for the evaluation of myocarditis compared to CMR alone, particularly in the early stages of inflammation, and to potentially differentiate active inflammation from scars; however, more large-center trials are needed. This modality is also limited by availability and cost [60,61]. CMR is an invaluable tool not only for diagnosis, but also for prognosis in acute myocarditis. 

### 3.4. CMR during the COVID-19 Pandemic

Cardiac MRI offers many advantages compared to other imaging modalities during the COVID-19 pandemic. The ability to non-invasively obtain comprehensive answers by multicomponent imaging in a single setting greatly reduces healthcare exposure and the probability of propagating infection. Newer rapid scanning protocols with shorter acquisition times will only improve these efforts [62,63,64]. In the acute setting, clinical management can be greatly altered with non-invasive evaluation of myocardial injury, for example, differentiating ACS from acute myocarditis. In a single imaging study, CMR can evaluate cardiac function, ischemic changes, myocardial tissue viability, and valvular function with gold standard efficacy, providing an invaluable resource for diagnosis and risk stratification [65,66,67,68].

In the pandemic setting, most CMR programs have focused only on urgent studies that may alter acute patient care while deferring other less urgent studies in order to conserve personal protective equipment and other hospital resources that may be in shortage during this crisis. Appropriate CMR use guidelines have been released in concordance with society recommendations for the treatment of the SARS-CoV-2 virus, as published in JACC by Zoghbi et al. These guidelines advise shortened, more focused CMR protocols to reduce long-term exposure and maximize the number of studies to be conducted, emphasize prioritizing studies that will alter acute patient management, and encourage the use of pharmacological stress CMR to risk-stratify for coronary artery disease. Furthermore, a high incidence of arrhythmia has been seen in COVID-19, with a study from Wuhan, China, reporting over 40% of patients with severe illness developing an arrhythmia. Atrial fibrillation has been the most commonly associated condition, with over 90% of COVID-19 arrhythmia cases [12,69]. Transesophageal echocardiogram (TEE), traditionally used to assess left atrial appendage thrombi or assist in pulmonary vein isolation procedures, comes with high risk of respiratory tract exposure. CMR imaging may be used in place of TEE by utilizing a combination of cine imaging, contrast-enhanced magnetic resonance angiography, and late gadolinium enhancement with long inversion time [70]. LGE has been shown to maintain high tissue contrast and reproducible results with rhythm irregularity.

Puntmann et al. published a prospective observational study showing a high burden of abnormal CMR findings in a relatively small series of recently “recovered” COVID-19 patients [55]. However, larger cohorts of patients with longer follow-up periods, likely from multiple studies, are needed before generalized interpretations and conclusions can be drawn. In that effort, there are currently several ongoing clinical trials involving COVID-19 patients, including an NIH-sponsored prospective observational cohort study evaluating the short- and long-term sequalae of the COVID-19 infection and the effects of therapeutics, using CMR for cardiac evaluation [71].

## 4. Conclusions

Myocardial injury is a common complication of the COVID-19 illness and is associated with a worsened prognosis. SARS-CoV-2-induced myocardial injury has been observed primarily in the systemic hyperinflammatory stage of the disease. While elevated biomarkers, dynamic ECG changes, and echocardiographic findings of ventricular dysfunction are associated with myocardial injury, CMR is the preferred imaging modality for non-invasive evaluation in acute myocarditis per the AHA and ESC guidelines [2,32,33,34,35,36]. The revised 2018 LLC reflect the diagnostic accuracy gained by using quantitative parametric mapping to complement traditional CMR evaluation of myocardial inflammation. In the COVID-19 pandemic setting, CMR has been shown to be especially advantageous for diagnosis and risk stratification of acute myocardial injury, with modified scanning protocols recommended to minimize exposure and optimize patient care. The use of CMR in clinical trials evaluating agents that target myocardial inflammation and its sequelae will enable investigators to visualize therapeutic effects previously not seen in non-CMR trials. Finally, the growing use of CMR in clinical practice to assess myocardial injury in the acute stage of illness and on follow-up will lead to an improved understanding of the long-term sequelae of chronic myocardial inflammation from various pathological etiologies, including COVID-19.

## Abbreviations

Coronavirus disease 2019	COVID-19
Cardiac magnetic resonance imaging	CMR
Early gadolinium enhancement	EGE
Late gadolinium enhancement	LGE
Electrocardiogram	ECG
Left ventricular ejection fraction	LVEF
Disseminated intravascular coagulation	DIC
Acute coronary syndrome	ACS
Transthoracic echocardiogram	TTE
Transesophageal echocardiogram	TEE
Endomyocardial biopsy	EMB
Lake Louise criteria	LLC
Ischemic cardiomyopathy	ICM
Non-ischemic cardiomyopathy	NICM
